# Transplant ethics under scrutiny – responsibilities of all medical professionals

**DOI:** 10.3325/cmj.2013.54.71

**Published:** 2013-02

**Authors:** Torsten Trey, Arthur L. Caplan, Jacob Lavee

**Affiliations:** 1 Executive Director, Doctors Against Forced Organ Harvesting, Washington, DC, USA; 2 Drs. William F and Virginia Connolly Mitty Chair, Director, Division of Medical Ethics, NYU Langone Medical Center, New York, NY, USA; 3 Tel Aviv University, Leviev Heart Center, Sheba Medical Center, Ramat Gan and the Sackler Faculty of Medicine, Tel Aviv, Israel

## Abstract

In this text, we present and elaborate ethical challenges in transplant medicine related to organ procurement and organ distribution, together with measures to solve such challenges. Based on internationally acknowledged ethical standards, we looked at cases of organ procurement and distribution practices that deviated from such ethical standards. One form of organ procurement is known as commercial organ trafficking, while in China the organ procurement is mostly based on executing prisoners, including killing of detained Falun Gong practitioners for their organs. Efforts from within the medical community as well as from governments have contributed to provide solutions to uphold ethical standards in medicine. The medical profession has the responsibility to actively promote ethical guidelines in medicine to prevent a decay of ethical standards and to ensure best medical practices.

## The situation

Transplant medicine, along with its blessings for the health of the patient, is challenging the ethical limits in medicine as much as any other medical disciplines. Like in other treatments, a “substance” is added to the human body: a pharmaceutical drug, an artificial joint, a dental crown etc. However, in allotransplantation, the “substance” added–a human organ – requires the prior donation of an organ. In the case of living organ donation, the recipient depends on the voluntary, altruistic donation of a suitable organ by the donor. Yet, one might often forget that the donor puts his own life at higher risk through his generosity. In the case of organ donation from the deceased, the donor has already lost his life before actually donating. In both cases, the altruistic decision to donate one’s organs is based on free, voluntary, and informed consent. Altruism and consent are requirements for ethical organ donation. This has been acknowledged as key requirements for organ donation in modern times in order to prevent against deception or coercion leading to organ donation (1).

The medical profession's mission is to either save people’s lives or improve their health. Transplant medicine walks a narrow line between saving a life and jeopardizing it in that it relies on risky surgery and powerful but dangerous medications. The establishment of strict ethical standards for obtaining organs, has allowed transplant medicine to navigate this path.

## Challenges in transplant medicine

In recent times, transplant medicine encountered a challenge – a severe shortage of organs. Sadly, organ trafficking and transplant tourism emerged in response to this challenge. Organ donation started to take a commercial route. Donors were persuaded, sometimes lured, and even coerced to donate a kidney for cash. Altruism is ignored in sales but what about consent?

Some say there is still voluntary consent, when people decide to sell their organs for money, but is it really a free decision? Usually the decision to commercially donate a kidney is dictated by the financial distress of the organ provider who desperately hopes to improve his financial situation likely not being aware that he possibly jeopardizes his health situation, which may add another financial burden. The financial coercion on the potential seller makes it insufficient to call it a free decision especially if there are no other choices open to acquire money.

There is a vicious circle at play. A simple cross-check on the so-called voluntary and free consent of a commercial organ sale is: would the seller still make his kidney available and expose his life to some higher risk if a monetary fund would offer the same amount of money to the potential seller as they would earn without selling an organ? One may assume that in the latter scenario all of the commercial sales would halt immediately. Commercial organ trafficking has taken transplant medicine to a troubling moral gray zone, and it is one of the transplant medicine’s responsibilities to prevent more severe moral problems from happening. Tolerating violations of medical ethics will result in further such violations.

We can observe the moral consequences of organ trafficking as it recently came to public awareness when physicians in German transplant departments manipulated patient data to achieve a higher organ recipient priority for their patients (2). Transplant medicine should have great interest to correct and prevent this phenomenon, because organ donors - the basis for allotransplant medicine – lose motivation to donate their organs as in the case of Germany where organ donations dropped by 12.8% in 2012 (3). Either with or without monetary payments, it is an inverse form of organ trafficking in which a medical doctor manipulated the priority criteria, thereby ranking would-be organ recipients intentionally closer to receiving organs. It is inverse in that the recipient is moved closer to the organ donor. This proceeding omits those recipients who suppose to receive the organs based on the regular waiting times, hence possibly jeopardizing the lives of the regular recipients. This inverse form of organ trafficking is a desperate attempt to accelerate the allocation of a transplant organ by omitting the ethically established pathway of the public organ distribution, which is based on altruistic organ donation and a transparent, traceable organ distribution system as outlined by the World Health Organization (WHO) (4).

While in the first case of organ trafficking monetary reimbursement and possibly a lack of scrutiny from medical doctors is involved, the second case of the inverse organ trafficking is based on an intentional breach with the ethically established pathway of organ distribution. Both practices lead transplant medicine into a regrettable moral gray zone. This gray zone gets even darker, when looking at the organ procurement practices in China, where a national law in 1984 allowed removing organs from executed prisoners for the purpose of transplantation. It was an unprecedented situation where executions and organ procurement from executed prisoners took place in the same country. Until today, it is unparalleled in the world that thousands of organs are procured each year in a fashion that is condemned by ethical standards of all major medical organizations (5,6).

First public awareness on this issue was raised when in 2001 Dr Wang Guoqi, one of the Chinese doctors who removed organs from executed prisoners, testified before US Congress. To be clear: the Chinese Criminal Code in article 211 demands that the execution is carried out within 7 days after the death sentence, and while the convict might still be under shock, Chinese authorities claim that death row candidates are still being asked to give consent for organ donation before the actual organ harvesting takes place after the execution. This proceeding defies all ethical guidelines that require a voluntary, free and informed consent. With a rope around the neck, a voluntary consent turns into an absurd mockery. While in Germany some transplant surgeons have left the ethical pathway of the public organ distribution system, in China, medical doctors have severely violated ethical principles by acquiring coerced “consent” prior to organ harvesting. Instead of organ donation, we rather choose organ harvesting to describe the organ procurement process. In this situation, transplant medicine in China has gone beyond the gray zone through the organ harvesting from executed prisoners, which under a different name is nothing else than killing for organs, an open contradiction to the mission of medicine.

According to official statements, 90% of the transplant organs from deceased donors in China stem from executed prisoners (7). The organ harvesting from executed prisoners in communist China incorporates the danger of a self-service organ procurement system. For 50+ reasons people can be sentenced to death, and one faces the question if transplant medicine benefits from the “legal system” or if transplant medicine actually influences jurisdiction, especially if a convict would provide the matching organ (8).

Transplant medicine in China has then eventually crossed the gray zone and took medicine into a black zone when after 1999 living people, detained prisoners of conscience, were systematically examined, blood tested, and categorized for transplantation and their organs harvested on-demand. Such “on-demand organ harvesting system” was based on a large pool of detained prisoners that allowed Chinese hospitals to advertise to offer any transplant organ within two weeks, a mystery as there was and still is no public organ donation program in China. The situation turned even more absurd when in 2006 the investigation report by David Matas and David Kilgour (9) unveiled that practitioners of the persecuted self-cultivation practice Falun Gong (10) were not only subject to torture, but also main target for the medical examinations while in detention, an implausible combination of opposite treatment procedures: torture and health care. The underlying crime against humanity appears as unbelievable as it is disturbing. One might be tempted to rather not believe it, as to avoid the outrage that one would otherwise feel. Yet, when Kilgour and Matas called 17 hospitals across China in 2006, the medical doctors in the Chinese hospitals admitted that they use “fresh” organs from Falun Gong practitioners. And when the WOIPFG called Dr Chen Rongshan in spring 2012, he admitted that the court had arranged the organ harvesting from detained Falun Gong practitioners (11).

Transplant medicine in China has ignored internationally acknowledged ethical principles and thereby challenged the medical profession in its entirety. Can the medical community remain silent when the ethical principles are violated to the degree that it basically turns the hippocratic ‘do no harm’ into the opposite? By not speaking up against it, we jeopardize our own position toward the code of ethics, and we passively grant permission for the next violation of medical ethics. When a medical colleague systematically commits malpractice, we would feel the need to bring it up, when a new pharmaceutical drug causes adverse effects, we feel obliged to halt its usage immediately and not after 5 years, yet, when in China living people are systematically killed for their organs, the medical community remains silent and is satisfied that they will halt the practice sometime in the future. The organ harvesting from living detained prisoners of conscience, such as in the case of Falun Gong, challenges our claim to respect bioethics. China’s abuse of transplant medicine is actually a chance for us to learn and to provide even better medicine. In September 2012, Dr Gabriel Danovitch said before US Congress “unethical medicine is bad medicine” (12,13). Promoting ethics in medicine, and especially in the field of transplant medicine, is the best way to provide the best medicine to our patients.

## What has been done, and what else can be done?

The medical community has the right and the responsibility to reject research papers that are based on unethical organ procurement practices. Following ethical standards in research might be challenging, but it will help us to stay on the right track. A prisoner who died from a gunshot or lethal injection is not the appropriate organ source to build scientific knowledge for future transplant therapy. One cannot exclude the possibility that the forced organ harvesting from executed prisoners and detained prisoners of conscience in China has found its way into research papers from China. This has provoked a reaction from the medical community in the form of a call to boycott Chinese research papers related to transplant medicine (14,15).

After the unethical organ harvesting from detained prisoners of conscience in China came to broader public awareness in 2006, a strong response from within the medical community was needed to safeguard ethical standards in medicine against being undermined. Consequently, medical doctors founded the medical advocacy group Doctors Against Forced Organ Harvesting (DAFOH) (16). DAFOH seeks to inform medical doctors and the public about forced organ harvesting and calls for an end to this practice. Increased information has also contributed to the publication of the book State Organs-Transplant Abuse in China (17). In the fall of 2012, DAFOH initiated petitions in Europe, Australia and the United States against China’s unethical organ harvesting, asking for further investigation through the United Nations Human Rights Council. The petitions generated together more than 250 000 signatures within 3 months, which indicates that the public has great interest in the medical profession upholding its ethical standards. This is also reflected by the increased interest from legislative bodies. In September and December 2012, the US Congress hosted two hearings on China’s unethical organ harvesting practice, and the European Parliament hosted a separate hearing in December.

As early as 2008, the Israeli Parliament adopted a new Organ Transplant Law banning the reimbursement of any transplant operation performed abroad if the operation is performed against local law or is associated with organ trade. Consequently, the flow of Israeli patients to China for new organs came to an abrupt and complete halt (18). Other parliaments are discussing next steps. It is our responsibility as medical doctors to safeguard ethical standards in medicine, which in the case of China includes the necessity to call for international investigation on forced organ harvesting from detained Falun Gong practitioners and other prisoners of conscience. Any step to promote medical ethics will be beneficial to the medical profession and our patients.
